# 异基因造血干细胞移植治疗DEK-NUP214融合基因阳性急性髓系白血病患者12例疗效分析

**DOI:** 10.3760/cma.j.cn121090-20230913-00115

**Published:** 2024-04

**Authors:** 昱妍 申, 栋林 杨, 祎 何, 爱明 庞, 欣 陈, 巧玲 马, 荣莉 张, 嘉璘 魏, 卫华 翟, 明哲 韩, 尔烈 姜, 四洲 冯

**Affiliations:** 1 中国医学科学院血液病医院（中国医学科学院血液学研究所），血液与健康全国重点实验室，国家血液系统疾病临床医学研究中心，细胞生态海河实验室，天津 300020 State Key Laboratory of Experimental Hematology, National Clinical Research Center for Blood Diseases, Haihe Laboratory of Cell Ecosystem, Institute of Hematology & Blood Diseases Hospital, Chinese Academy of Medical Sciences & Peking Union Medical College, Tianjin 300020, China; 2 天津医学健康研究院，天津 301600 Tianjin Institutes of Health Science, Tianjin 301600, China

## Abstract

2016年11月至2022年8月期间，12例DEK-NUP214融合基因阳性急性髓系白血病患者在中国医学科学院血液病医院接受异基因造血干细胞移植（allo-HSCT）。12例患者中男5例，女7例，中位年龄34（16～52）岁。12例患者初诊时均检出DEK-NUP214融合基因阳性，10例患者染色体核型分析显示t（6;9）（p23;q34）易位（2例患者未行染色体核型分析），11例患者检出FLT3-ITD突变，11例患者检出WT1高表达。9例患者allo-HSCT前处于第一次完全缓解状态，1例患者疾病未缓解，2例患者处于复发状态。所有患者均接受清髓性预处理，5例患者接受同胞全相合造血干细胞移植，7例患者接受单倍体造血干细胞移植。移植物单个核细胞中位数为10.87（7.09～17.89）×10^8^/kg，CD34^+^细胞中位数为3.29（2.53～6.10）×10^6^/kg。所有患者均获得造血重建，粒细胞植入中位时间为14（10～20）d，血小板植入中位时间为15（9～27）d。移植后1年移植相关死亡率为21.2％。移植后1、3年累积复发率分别为25.0％、50.0％，无白血病生存率为（65.6±14.0）％，总生存率为（72.2±13.8）％。

## Abstract (en)

Twelve DEK-NUP214 fusion gene-positive patients with acute myeloid leukemia and on allo-HSCT treatment at the Hematology Hospital of the Chinese Academy of Medical Sciences from November 2016 to August 2022 were included in the study, and their clinical data were retrospectively analyzed. The patients comprised five men and seven women with a median age of 34（16–52）years. At the time of diagnosis, all the patients were positive for the DEK-NUP214 fusion gene. Chromosome karyotyping analysis showed t（6;9）（p23;q34）translocation in 10 patients（two patients did not undergo chromosome karyotyping analysis）, FLT3-ITD mutation was detected in 11 patients, and high expression of WT1 was observed in 11 patients. Nine patients had their primary disease in the first complete remission state before transplantation, one patient had no disease remission, and two patients were in a recurrent state. All patients received myeloablative pretreatment, five patients received sibling allogeneic hematopoietic stem cell transplantation, and seven patients received haploid hematopoietic stem cell transplantation. The median number of mononuclear cells in the transplant was 10.87（7.09–17.89）×10^8^/kg, and the number of CD34^+^ cells was 3.29（2.53–6.10）×10^6^/kg. All patients achieved blood reconstruction, with a median time of 14（10–20）days for neutrophil implantation and 15（9–27）days for platelet implantation. The 1 year transplant-related mortality rate after transplantation was 21.2％. The cumulative recurrence rates 1 and 3 years after transplantation were 25.0％ and 50.0％, respectively. The leukemia free survival rates were（65.6±14.0）％ and（65.6±14.0）％, respectively. The overall survival rates were（72.2±13.8）％ and（72.2±13.8）％, respectively.

DEK-NUP214融合基因阳性急性髓系白血病（AML）在WHO（2017）分类中已明确归为特定类型，这类患者在AML中占1％～2％[1]。1976年首次报道染色体t（6;9）易位可形成DEK-NUP214融合基因[2]。其中DEK存在于染色体6p22，NUP214（也称为CAN）存在于染色体9q34[2]。既往研究表明伴t（6;9）易位的AML更常见于年轻患者，对化疗不敏感，且具有更差的预后[3]。既往研究表明，42％～90％的伴t（6;9）易位AML患者可检出FLT3-ITD基因突变阳性[1],[4]–[6]。伴t（6;9）易位的儿童、成人AML患者5年总生存（OS）率分别为28％、9％[1],[4]。在2023年NCCN指南中，伴该染色体易位的AML患者明确归为预后不良组[7]。国外一项多中心研究结果显示，89例伴t（6;9）易位成人AML患者在第1次完全缓解（CR1）期接受异基因造血干细胞移植（allo-HSCT），移植后5年OS率、无白血病生存（LFS）率分别为53％、45％[4]。本研究对在本中心接受allo-HSCT的12例DEK-NUP214阳性AML患者进行回顾性分析，评估allo-HSCT对DEK-NUP214阳性AML的疗效。

## 病例与方法

一、病例特征

本项回顾性研究纳入2016年11月至2022年8月在中国医学科学院血液病医院接受allo-HSCT的12例DEK-NUP214融合基因阳性AML患者。男5例，女7例，中位移植年龄34（16～52）岁。12例患者初诊时均检出DEK-NUP214融合基因阳性，10例患者染色体符合t（6;9）（p23;q34）易位，2例患者无染色体核型分析结果；11例患者初诊时检出FLT3-ITD基因突变，11例患者伴WT1基因高表达，1例患者未检出FLT3-ITD突变，1例患者不伴WT1高表达。1例患者起病时有骨髓外浸润，但在移植时处于完全缓解（CR）状态。3例患者在移植前曾接受FLT3-ITD抑制剂索拉非尼（2例）和吉瑞替尼（1例）治疗，这3例患者中2例移植前为微小残留病（MRD）阴性。起病时无FLT3-ITD突变的1例患者，检出CBL基因exon9突变、KMT2D基因exon48突变和WT1基因高表达。患者临床特征详见表1。

**表1:** 接受异基因造血干细胞移植治疗的12例DEK-NUP214融合基因阳性AML患者的临床特征

例号	性别	年龄（岁）	起病染色体核型	其他合并基因突变
1	女	28	46,XX,?t(6;9)(p23;q34)[20]	FLT3-ITD突变；WT1高表达；CSF3R基因P733T突变；CRLF2基因P224L突变；WAS基因R268Q突变；CYLD基因E720fs突变；SETBP1基因S1287L突变
2	女	39	46,XX,t(6;9)(p22;q34)[20]	FLT3-ITD突变
3	男	18	46,XY,t(6;9)(p22;q34)[20]	FLT3-ITD突变；WT1高表达
4	女	16	46,XX,t(6;9)(p23;q34)[2]/46,XX[18]	FLT3-ITD突变；WT1高表达
5	女	44	46,XX,t(6;9)(p23;q34.1)[20]	WT1高表达；FLT3-ITD突变；EZH2 p.y733fs突变；WT1 exon7突变
6	男	46	/	FLT3-ITD；WT1高表达
7	男	29	46,XY,t(3;12)(p21;p13),t(6;9)(p23;q34)[18]/46,XY[2]	FLT3-ITD突变；WT1高表达；WT1 exon 7突变；NOTCH1 exon 14突变；PDS5B exon 19突变；PHF6 exon 4突变
8	女	48	/	CBL exon 9突变；KMT2D exon 48突变；WT1高表达
9	女	52	46,XX,t(6;9)(p23;q34)	FLT3-ITD突变；WT1高表达
10	男	17	46,XY,t(6;9)(p23;q34.1)[20]	WT1高表达；FLT3-ITD突变
11	女	20	46,XX,t(6;9)(p23;q34)[20]	WT1高表达；FLT3-ITD高表达
12	男	40	46,XY,t(6;9)(p23;q34)[20]	FLT3-ITD突变；WT1高表达；SF3B1 exon 14突变；PlCG1 exon 1突变；FAT1 exon 10突变

**注** AML：急性髓系白血病；/：无数据

二、移植时病情及移植特征

1例患者初诊为骨髓增生异常综合征（MDS）伴原始细胞增多2型（MDS-EB2），未接受化疗，因转为AML而接受造血干细胞移植，其余患者均以AML起病。移植前接受化疗的11例患者中，获得CR1的中位疗程数为1（1～3）个，其中6例患者诱导治疗后达CR，3例患者2个疗程化疗后达CR，3例患者3个疗程化疗后达CR。移植前所有患者接受中位4（2～6）个疗程化疗，疾病确诊至移植的中位时间为6.5（3～12）个月。9例患者移植前原发病处于CR1状态，1例患者疾病未缓解，2例患者处于复发状态。5例患者接受同胞全相合造血干细胞移植，7例患者接受单倍体造血干细胞移植。所有患者均接受清髓性预处理，回输物均为外周血造血干细胞。5例患者接受白消安+环磷酰胺+氟达拉滨/克拉屈滨+阿糖胞苷为基础的预处理方案，3例患者接受白消安+氟达拉滨+去甲氧柔红霉素+环磷酰胺预处理方案，2例患者接受白消安+美法仑+环磷酰胺±克拉屈滨方案，1例患者接受全身放射治疗（TBI）+克拉屈滨+阿糖胞苷+环磷酰胺预处理方案，1例患者接受白消安+氟达拉滨+阿糖胞苷预处理方案。所有患者以环孢素A或他克莫司预防急性移植物抗宿主病（aGVHD）。单倍体移植的患者在预处理中加用兔抗人胸腺细胞免疫球蛋白（rATG），移植后加用霉酚酸酯，在移植后1、3、6、11 d加用小剂量甲氨蝶呤[8]–[9]。移植物中位单个核细胞数为10.87（7.09～17.89）×10^8^/kg，CD34^+^细胞为3.29（2.53～6.10）×10^6^/kg。详见表2。

**表2:** 12例DEK-NUP214融合基因阳性急性髓系白血病患者移植特征及转归

例号	预处理方案	供者	移植时疾病状态	回输单个核细胞（×10^8^/kg）	回输CD34^+^细胞（×10^6^/kg）	移植相关死亡	粒细胞植入（d）	血小板植入（d）	复发（含分子学复发）	随访时间（d）	结局
1	Bu+Flu+Ara-C	同胞全相合	CR1	7.48	3.21	否	13	16	否	1096	存活
2	TBI+Clad+Ara-C+Cy	同胞全相合	CR1	10.75	3.97	否	20	19	否	503	存活
3	Bu+Flu+Ara-C+Cy	同胞全相合	CR1	7.09	3.54	否	12	12	否	309	存活
4	Bu+Cy+Clad+Ara-C	同胞全相合	复发	10.00	2.70	否	13	14	是	112	死亡
5	Bu+Mel+Cy	同胞全相合	复发	14.90	3.72	否	12	11	否	256	存活
6	Bu+Flu+IDA+ATG+Cy	单倍体	CR1	8.72	4.09	否	14	12	是	1479	死亡
7	Bu+Cy+Clad+Ara-C+ATG	单倍体	CR1	11.63	2.90	否	15	15	是	579	存活
8	DAC+Bu+Ara-C+CLad+Cy+ATG	单倍体	NR	17.82	3.36	否	12	12	否	1100	存活
9	Bu+Flu+IDA+Cy+ATG	单倍体	CR1	13.00	2.60	否	14	25	是	182	存活
10	Mel+Clad+Bu+Cy+ATG	单倍体	CR1	10.00	6.10	否	18	27	否	326	存活
11	Bu+Flu+IDA+Cy+ATG	单倍体	CR1	11.00	2.53	是	18	19	否	205	死亡
12	Bu+Clad+Ara-C+Cy+ATG	单倍体	CR1	13.69	3.01	是	10	9	否	300	死亡

**注** CR1：第一次完全缓解；NR：未缓解；Bu：白消安；Flu：氟达拉滨；Ara-C：阿糖胞苷；TBI：全身放射治疗；Clad：克拉屈滨；Cy：环磷酰胺；Mel：美法仑；ATG：抗人胸腺细胞球蛋白；IDA：去甲氧柔红霉素

三、随访及定义

采用查阅门诊/住院病历和电话联系的方式进行随访。OS、LFS均从造血干细胞回输之日开始计算。MRD阳性定义为无血液学复发的DEK-NUP214融合基因阳性或流式细胞术检测阳性。随访截止日期为2023年5月26日。

四、统计学处理

LFS和OS计算采用Kaplan-Meier法。所有统计通过SPSS 22.0软件计算。

## 结果

一、造血重建及移植物抗宿主病

所有患者均获得造血重建，中性粒细胞中位植入时间为14（10～20）d，血小板植入中位时间为15（9～27）d。

在移植0 d时，3例患者流式细胞术MRD阳性，其余患者均为MRD阴性。1例患者单倍体移植后205 d死于aGVHD（供者为父亲），1例患者单倍体移植后299 d死于感染引起的继发移植物排斥（同胞供者）。移植后1年预估移植相关死亡率为21.2％。所有患者中，3例出现Ⅲ/Ⅳ度aGVHD，死亡1例，其余2例患者均治愈。

二、复发及转归

12例患者中有4例移植后出现MRD阳性，1例患者移植前至死亡MRD均未转阴，移植后112 d死于白血病。其余3例患者移植后复发中位时间为182（98～703）d。至随访截止日期，仅1例患者存活，该患者在移植后98 d复发，复发后接受伊达比星加柔红霉素化疗后回输供者造血干细胞后获CR，后予索拉非尼、阿扎胞苷、地西他滨维持治疗，至随访截止日期，移植后无白血病生存579 d，其余复发患者均死于白血病。移植后1、3年累积复发率分别为25.0％、50.0％。

三、生存分析

所有患者移植后1、3年的LFS率为（65.6±14.0）％，OS率为（72.2±13.8）％。至随访截止日期，8例患者存活，其中7例起病时伴FLT3-ITD突变阳性，移植后有3例患者接受了FLT3-ITD抑制剂维持治疗（索拉非尼2例，吉瑞替尼1例），1例患者接受了阿扎胞苷维持治疗。移植前为复发或未缓解状态的3例患者中，1例患者因复发死亡，1例患者复发治疗后达CR，1例患者至随访末期无白血病生存。本研究中唯一无WT1高表达的患者起病时伴齿龈髓外浸润，但移植后持续缓解，至随访截止日期，移植后无病生存503 d。本研究中无FLT3-ITD突变的MDS-AML的患者，至随访截止日期，移植后无病生存1 100 d。

## 讨论

染色体t（6;9）（p23;q34）易位可见于AML和MDS[10]。而无论起病时诊断为AML还是MDS，该类患者均预后不良，但allo-HSCT可改善这类患者的预后[11]。1976年研究证实t（6;9）（p23;q34）易位在分子学基础上可形成DEK-NUP214融合基因[2]。DEK是一种原癌基因，在AML细胞中表达增加[12]。NUP214基因又被称为CAN基因，在急性白血病中除了与DEK基因形成融合基因外，也可与SET基因形成SET-NUP214基因，见于急性T淋巴细胞白血病和AML，NUP214也与ABL基因融合（见于急性淋巴细胞白血病）[13]。既往多项研究显示，DEK-NUP214融合基因阳性常伴随FLT3-ITD突变，与FLT3-TKD突变同时发生则不常见[3],[5]。但DEK-NUP214融合基因对于白血病的影响并不是通过影响FLT3-ITD来完成，而可能通过影响mTOR和STAT5信号通路[14]。

t（6;9）易位或DEK-NUP214阳性在多项指南中均归为预后不良的染色体异常，AML伴DEK-NUP214融合基因在WHO（2022）分类中归为特殊类型AML，与其他AML患者相比，这类患者相对年轻化，中位发病年龄为23～44岁[15]–[16]。与本研究中位发病年龄34岁基本一致。既往研究表明，伴t（6;9）易位的AML患者的CR率不超过50％，若不接受移植，中位生存期为诊断后1年[17]–[18]。故在CR期行造血干细胞移植为这类患者的首选方案[4]。2019年一项大系列国际多中心研究中，178例伴t（6;9）的AML患者入组，伴FLT3-ITD突变患者占62％，81％的患者获得CR，5年OS率为38％（95％*CI* 31％～47％），其中66％的患者接受allo-HSCT；在CR1期行移植的患者5年OS率（53％）优于化疗患者（23％），化疗患者5年无病生存率仅为7％，在CR1、CR2期和非CR状态下行移植的患者移植后生存无明显差别，其OS率与本组病例相似。FLT3-ITD突变在这个研究队列中显示对预后无影响[4]，2014年一项儿童AML的研究也证实合并FLT3-ITD患者不具有更差的预后[5]。而在本研究中，移植前处于非CR状态的3例患者中有2例在末次随访时保持无病生存状态，提示强行移植的患者仍有望获得较好的移植后生存。既往有研究显示具有t（6;9）易位患者与正常染色体核型患者相比，allo-HSCT后的OS和LFS差异无统计学意义[19]。

伴DEK-NUP214融合基因的MDS的患者能否归为AML，目前仍有争议，在WHO（2022版）分类中DEK-NUP214融合基因归为AML重现遗传学异常，而在一项纳入107例伴t（6;9）易位AML/MDS患者的研究中，MDS伴DEK-NUP214比AML伴DEK-NUP214的患者具有更低的白细胞、更低的FLT3-ITD合并检出率，但MDS和AML患者均预后较差，且两者差异无统计学意义[11]。本研究中在初诊时诊断为MDS-EB2的患者在移植前已转化为AML，未行化疗而直接行allo-HSCT，该患者移植后至末次随访仍为无病生存。至于这两类患者是否异质，需要更大样本的研究。但对于这两类患者来说，预后均不佳，且allo-HSCT均可提高疗效[11]。

既往研究表明，高表达WT1的AML患者更易发生诱导化疗失败和复发[20]。在本研究中观察到大部分DEK-NUP214阳性的AML患者起病时具有WT1高表达，由于例数较少，该基因异常是否影响DEK-NUP214阳性AML患者移植后生存尚待进一步研究。

本组病例结果显示，allo-HSCT治疗DEK-NUP214融合基因阳性AML可获得较好的疗效。
